# Sexual and reproductive health mobile apps: results from a cross-sectional values and preferences survey to inform World Health Organization normative guidance on self-care interventions

**DOI:** 10.1080/16549716.2020.1796346

**Published:** 2020-08-11

**Authors:** Carmen Logie, Moses Okumu, Heather Abela, David Wilson, Manjulaa Narasimhan

**Affiliations:** a Factor-Inwentash Faculty of Social Work, University of Toronto, Toronto, ON, Canada; b School of Social Work, University of North Carolina, Chapel Hill, NC, USA; c Health, Nutrition, and Population Global Practice, World Bank, Washington, DC, USA; d Department of Sexual and Reproductive Health and Research, World Health Organization, Geneva, Switzerland

**Keywords:** Sexual health, reproductive health, mHealth, healthcare provider, mobile app, healthcare training, access

## Abstract

Mobile application (app) platforms have the potential to advance sexual and reproductive health (SRH). Yet there is a dearth of knowledge regarding global perspectives from healthcare providers on how SRH mobile apps are being leveraged in their healthcare practice. In 2019 the World Health Organization (WHO) developed a consolidated guideline on self-care interventions for SRH. To inform this guideline, we conducted a global values and preferences survey. This study aimed to (a) understand the awareness, access, and uptake of SRH mobile apps; (b) examine how many healthcare provider (HCP) participants provided linkages, referrals and information to clients regarding SRH mobile apps; and (c) among HCP, assess how many felt confident and informed regarding SRH mobile apps. We hosted a cross-sectional web-based survey on the WHO Department of Reproductive Health and Research website and shared the survey with SRHR listservs. There were 825 survey participants, 360 whom identified as healthcare providers (HCP). Approximately one-third of HCP participants had provided a referral/information to their clients about sexual or reproductive health apps. While 40.8% of HCP felt confident and informed about sexual health apps, half (47.4%) reported needing more information, and 15.6% expressed interest in receiving training to use in practice. While 42.6% of HCPs felt confident and informed about reproductive health apps, 45.7% needed more information, and 15.1% were interested in further training. There was also an open-ended question for HCP to share their thoughts about self-care SRH interventions. Specifically regarding SRH apps, HCP responses revealed the importance of considering: (a) security and confidentiality; (b) potential benefits of SRH apps for underserved groups (i.e. youth, rural communities); (c) community engagement; (d) health benefits; and (e) and online training for HCP on SRH mobile apps. Findings signal interest and opportunities for training and engaging HCP in using mobile apps to advance SRH.

## Background

Understanding global perspectives from healthcare providers on access and uptake of digital technologies and mobile platforms provides insight into opportunities to advance sexual and reproductive health (SRH). Findings are presented from a global values and preferences survey (GVPS) [1] regarding SRH mobile applications (apps) that informed the 2019 World Health Organization (WHO) *Consolidated guideline on self-care interventions for health: sexual and reproductive health and rights* [2].

The *WHO Guideline Recommendations on Digital Interventions for Health System Strengthening* identified digital technologies as a way to increase universal health coverage and defines digital health interventions as ‘a discrete functionality of digital technology that is applied to achieve health objectives and is implemented within digital health applications and ICT systems’ [3]. Digital technologies were further delineated in the *WHO Classification of Digital Health Interventions* into client, healthcare provider, health system manager, and data services focused [4].

Digital technologies have the potential to facilitate innovative, efficient ways of meeting growing global health needs. A 2019 report [5] indicated that there are over 5 billion mobile phone users, 4 billion internet users, and 3 billion active social media users, with most accessing the internet through mobile devices. The anonymity, convenience, and accessibility afforded by short messaging services (SMS), web access, and video streaming enhance the potential for SRH interventions to reach persons disconnected from mainstream SRH services.

Governments are leveraging digital technologies to strengthen health systems to ensure record sharing and client monitoring [6]. Health innovators have developed myriad apps and other channels (e.g. SMS) to increase information access. A scoping review [6] identified that improved access, provision, and engagement with sexual health information and care were the primary benefits of digital sexual health communication interventions. A systematic review of mobile phone interventions for adolescent SRH reported that SMS health promotion campaigns contributed to improved SRH outcomes [7].

Applying digital technologies to SRH self care may increase SRH information and resources access [8]. For instance, for self-care interventions such as HIV self-testing [9], digital technologies could facilitate further support via mobile phones, instructional videos, or SMS. Thus, the use of digital technologies for SRH self care could improve correct use of interventions, and empower individuals to better manage their health [8]. Mobile apps for SRH may be used by lay persons for particular functions (e.g. acquiring information) and by healthcare providers (HCP) in numerous ways (e.g. consulting with remote clients) [4]. Yet there is limited global evidence regarding HCP’s knowledge and comfort with digital technologies such as mobile apps for promoting and/or using SRH self-care interventions (Table 1), or their training needs and preferences. Our research questions were: (a) across global regions, what is the awareness, access, and uptake of SRH mobile apps?; (b) among HCP, how many have provided linkages, referrals, and information to clients regarding SRH mobile apps?; and (c) among HCP, how many feel confident and informed regarding SRH mobile apps, and how many would like further information and training?

**Table 1.** Examples of sexual and reproductive health mobile applications for self care and relevance to the WHO classification of digital health interventions v1.0^4^.

Categories		Definitions
**Interventions for clients**: Clients are members of the public who are potential or current users of health services, including health promotion activities. Caregivers of clients receiving health services are also included in this group.		
**Client focused digital interventions relevant to sexual and reproductive health mobile apps for self care include:**		
1.1 Targeted client communication	1.1.1: health event alerts to specific populations (e.g. could relay STI outbreak)	
	1.1.2: targeted health information based on health status or demographics (e.g. could focus on same-sex practices among men)	
	1.1.3: targeted alerts and reminders to clients (e.g. for HIV and/or STI testing, medication adherence)	
	1.1.4: transmit diagnostic result or result availability to client (e.g. STI result is ready at nearby clinic)	
1.3 Client to client communication	1.3.1: Peer group for client (e.g. for antiretroviral therapy adherence among people living with HIV)	
1.4 Personal health tracking	1.4.3: Client can capture data (e.g. on adherence)	
1.6 On-demand information services to clients	1.6.1: Client retrieving health information (e.g. looking up HIV prevention strategies)	
**Healthcare provider focused digital interventions relevant to sexual and reproductive health mobile apps for self care include:**		
2.4 Telemedicine	2.4.1: Consultations between remote client and healthcare provider (e.g. about adherence, side effects)	
	2.4.3: Transmission of medical data to healthcare provider (e.g. self-testing results)	
2.8 Healthcare provider training	2.8.1: Provide healthcare providers training (e.g. how to assess helpful and accurate SRH mobile apps)	
	2.8.1: Assess capacity of healthcare provider (e.g. regarding SRH knowledge and needs for specific populations, for instance lesbian, gay, bisexual and transgender persons)	

## Methods

The GVPS was a self-administered online cross-sectional survey, approximately 20 minutes in duration, conducted between July 2018 and November 2018 in English, Spanish and French. Inclusion criteria were being aged 18 and over, able to read one of the survey languages, and able to provide informed consent. Research Ethics Board approval was attained from the University of Toronto. Participant recruitment was conducted by hosting the survey on the WHO Department of Reproductive Health and Research website [10] and sharing the survey link with diverse (>35) SRHR listservs. To reduce response bias, surveys were anonymous and questions were optional. We aimed to have 1000 participants across countries to achieve diversity in respondents regarding gender, age, and regional representation. Study details can be found in the GVPS report [1].

There were questions for all participants and a sub-set for self-identified HCP. We asked all participants awareness and usage questions regarding sexual health (SH) and reproductive health (RH) apps: (1) *‘have you heard of’* (responses: I don’t know what that is; yes, but I don’t know how to access; yes, I know what this is, and I know where to access), (2) *‘have you used’* (responses: I don’t need this/not relevant; no, I have not used; yes, I have used in the past three months; yes, I have used), and (3)*‘what are the top factors for deciding to use’* (responses: privacy and confidentiality, lack of judgment, empowerment, confidence, accessibility). We also asked HCP respondents their experience providing referrals/information regarding SH and RH apps (*‘have you provided a referral or information about these services to your patients/clients’*, responses: no, yes, it is not available where I live, it is not related to my job) and self-perceived confidence and information/training needs (*‘how confident and informed do you feel about these services’,* responses: I need training to provide this service, I need more information, I feel confident and informed). The survey also provided an opportunity for HCP participants to provide open-ended feedback regarding SRH self-care interventions (*Is there anything you would like to share with us?*).

## Results

There were 825 participants: 360 participants identified as HCP and 465 participants did not identify as HCP (lay persons). Table 1 details participant socio-demographic characteristics. Locations of residence across WHO regions and participant age were diverse (see Table 2). Full details regarding survey implementation and participants may be found in the GPVS report [1]. Responses are detailed in Table 3 and summarized below.

**Table 2.** Sociodemographic background of values and preferences survey respondents.

	Healthcare Providers (n = 360)^	Lay Person Respondents* (n = 465)^	Total
**Gender**			
Woman	248 (68.9%)	316 (68.0%)	564
Man	111 (30.8%)	140 (30.1%)	251
Transgender	1 (0.3%)	6 (1.3%)	7
Prefer not to say	0 (0%)	3 (0.6%)	3
Subtotal	360 (100%)	465 (100%)	825
**Age**			
	358 (100%)	464 (100%)	822
*Mean*	*38.0 (SD: 13.6)*	*31.9*	*34.6*
*Median*	*35.5*	*27.0*	*31*
**Region**			
African Region	102 (28.3%)	89 (19.1%)	191
Region of the Americas	125 (34.7%)	109 (23.4%)	234
South-East Asia Region	22 (6.1%)	20 (4.3%)	42
European Region	84 (23.3%)	157 (33.8%)	241
Eastern Mediterranean Region	17 (4.7%)	37 (8.0%)	54
Western Pacific Region	10 (2.8%)	53 (11.4%)	63
Subtotal	360 (100%)	465 (100%)	825
**Sexual Orientation**			
Heterosexual/Straight	304 (84.9%)	351 (75.6%)	655
Sexually Diverse (LGBQ+)	48 (13.4%)	103 (22.2%)	151
Prefer not to say	6 (1.7%)	10 (2.2%)	16
Subtotal	358 (100%)	464 (100%)	822
**Size of City/Town**			
A big city (above 1 million inhabitants)	177 (49.6%)	83 (49.7%)	260
A large city (300,000–1 million inhabitants)	66 (18.5%)	32 (19.2%)	98
A city (100,000–300,000 inhabitants)	39 (10.9%)	14 (8.4%)	53
Large town (20,000–100,000 inhabitants)	40 (11.2%)	15 (9.0%)	55
Town (1000–20,000 inhabitants)	25 (7.0%)	16 (9.6%)	41
Small town or hamlet (less than 1000 inhabitants)	10 (2.8%)	7 (4.2%)	17
Subtotal	357 (100%)	167 (100%)	524
**Highest Level of Education**			
Completed high school	24 (6.7%)	49 (27.5%)	73
A university bachelor’s degree	97 (27.1%)	65 (36.5%)	162
A graduate degree	235 (65.6%)	63 (35.4%)	298
Other	2 (0.6%)	1 (0.6%)	3
Subtotal	358 (100%)	178 (100%)	536
**Type of healthcare provider**			
Doctor	98 (27.6%)		
Pharmacist	80 (22.5%)		
Work in clinic/agency that provides SRHR information or education	78 (22.0%)		
Other (common responses for other: public health professional, student)	70 (19.7%)		
Public health activist	66 (18.6%)		
Health educator	54 (15.2%)		
Nurse or other healthcare professional	42 (11.8%)		
Community worker	23 (6.5%)		
Midwife	11 (3.1%)		

*Respondents who did not report being healthcare providers.

^responses were voluntary, so figures may not equal total participant numbers due to missing responses.

**Table 3.** Awareness, uptake, and training needs for sexual and reproductive health mobile apps from participants in a global values and preferences survey.

	Sexual health mobile app		Reproductive health mobile app	
Question	Healthcare provider	Lay person	Healthcare provider	Lay person
***Awareness***				
Aware of and where to access it	169 (47.3%)	176 (41.7%)	199 (56.1%)	226 (53.8%)
Aware of but not where to access	105 (29.4%)	117 (27.7%)	95 (26.8%)	96 (22.9%)
Not aware of	83 (23.2%)	129 (30.6%)	61 (17.2%)	98 (23.3%)
***Usage***				
Have ever used	42 (12.2%)	46 (12.7%)	60 (17.6%)	74 (20.4%)
Have used in past 3-months	12 (3.5%)	10 (2.8%)	24 (7.0%)	10 (2.8%)
Never used	227 (66.0%)	219 (60.3%)	195 (57.2%)	185 (51.1%)
***Top factors for deciding to use***				
Privacy and confidentiality	110 (41.0%)	65 (42.2%)	109 (40.8%)	65 (42.5%)
Lack of judgment	61 (22.8%)	30 (19.5%)	58 (21.7%)	29 (19.0%)
Empowerment	78 (29.1%)	37 (24.0%)	83 (31.1%)	38 (24.8%)
Convenience	134 (50.0%)	71 (46.1%)	135 (50.6%)	70 (45.8%)
Accessibility	134 (50.0%)	83 (53.9%)	134 (50.2%)	83 (54.2%)
**Healthcare provider perspectives**				
***Ever provide a referral/information***				
Yes	103 (34.3%)		106 (35.2%)	
No	123 (41.0%)		120 (39.9%)	
Not available where I live	16 (5.3%)		17 (5.6%)	
Unrelated to my job	60 (20.0%)		60 (19.9%)	
***Information/training needs***				
Feel confident and informed	118 (40.8%)		124 (42.6%)	
Need more information	137 (47.4%)		133 (45.7%)	
Need more training	45 (15.6%)		44 (15.1%)	

### Sexual health (SH) mobile apps

Less than half of participants (HCP: 47.3%; lay persons: 41.7%) reported that they knew what SH apps were and how to access; approximately one-tenth reporting ever using them (HCP: 12.2%; lay persons: 12.7%). Approximately one-third (34.3%) of HCP participants had provided a referral/information to their clients about SH apps. HCP that reported they or their partner had ever used a SH app were more likely to have referred an SH app to their clients (chi-square (1, n = 292) = 41.18, p < 0.0001). While 40.8% of HCP felt confident and informed about SH apps, nearly half (47.4%) reported needing more information and 15.6% training.

### Reproductive health (RH) mobile apps

Just over half of participants (HCP: 56.1%; lay persons: 53.8%) reported knowing what a RH app was and how to access, and approximately one-fifth (HCP: 17.6%, lay persons: 20.4%) had ever used one. While 42.6% of HCPs felt confident and informed about RH apps, 45.7% needed more information and 15.1% more training. One-third of HCP (n = 106; 35.2%) had provided a RH app referral/information to clients. HCP that reported that they or their partner had ever used a RH app were more likely to have referred an RH app to their client (chi-square (1, n = 289) = 21.92, p < 0.0001).

### Qualitative responses from healthcare providers

Table 4 reports qualitative responses by HCP participants about SRH apps. Themes included the importance of (a) security and confidentiality; (b) SRH apps in increasing access to information for underserved groups; (c) community engagement to increase SRH digital service awareness; (d) SRH apps for advancing health; and (e) recommendations for online SRH mobile apps training.

**Table 4.** Healthcare provider open-ended responses on sexual and reproductive health mobile apps and alignment with WHO classification of digital health interventions v1.0^4^ (n = 245).

Theme		Illustrative Quotation
Security and confidentiality		Concerns about cybersecurity although I don’t know enough about the risks. (US)
		(Concerns include) confidentiality of mobile apps or digital interventions (Switzerland)
		
Access	Benefits for youth	A reliable, up-to-date and simple to use online/mobile source of information would be highly beneficial, especially to younger populations. (Croatia) (1.61. client look up of information)
		Mobile application on SRH is very important currently for young people since everyone at least has a smartphone. (Uganda) (1.1.2 targeted health information to client based on demographics)
	Benefits for rural areas	Through social media, online app and for uneducated women in remote areas, we make use of mobile services (Nigeria) (1.1.2 targeted health information to client based on demographics)
		
Community engagement		Online mobile devices. Education for community leaders to mitigate sociological/cultural/religious factors. (US) (1.1.1 health event alerts, and 1.1.2 health information, to specific population groups)
		Community mobilization and sensitization is key to inform the communities about the services (RSA) (1.3.1 peer group for clients)
		
Linkage to healthcare		The mobile app on SRH should have facility names close to patient communities, community workers should equipped to rendered health care services in their various communities and referrals when necessary. (Nigeria) (2.4.2 remote monitoring of client health or diagnostic data by provider)
		With a list of health services in the area; an application for mobiles with this information. (Brazil) (1.6.1 client look-up of health information)
		Provide app, online services and information, ideally for free, including treatment and follow-up. (Mexico) (1.1.2 targeted health information to client based on health status/demographics)
		In the app, there is a constant reminder for the patient to visit a healthcare provider and log the details in the app with follow ups. (Kenya) (1.1.3 targeted alerts and reminders to clients)
		With a cell phone application, a page on the internet where you enter your test result or with a chip to detect the result and upload it. (Brazil) (1.4.2 self monitoring of diagnostic data)
		
Considerations	Vulnerable may have limited access to internet	Does not work if the person doesn’t have a computer, smart phone or regular internet access. Vulnerable populations struggle to use online interventions regardless if they are free (UK)
		An online platform so that those who are afraid to go to the clinic can get right medical attention and for those who can’t access internet, give a free toll number or rather employ nearby health workers in the community the client is. (Kenya) (2.4.1 consultation between remote client and healthcare provider)
		
Training needs (aligns with 2.8: Healthcare Provider Training, 2.8.1 training content to healthcare providers)		Training on mobile app usage in rendering services. (Ghana)
		Online training courses, links to valid sources of information like the WHO (Kenya)
		Training on self testing kits and how to use reproductive health mobile apps (Kenya)
		Online tutorial by healthcare providers (UK) and online workshops (Uruguay)

## Discussion

Overall, GVPS findings signal widespread awareness of SRH health apps, but lower uptake. As HCP reported interest in providing clients with SRH app information, it is important to address barriers to their referrals to clients. We also report valuable information on HCP training needs and preferences for integrating SRH apps into their practice. As HCP’s use of SH/RH apps was associated with a much higher likelihood of client referrals, HCP trainings could involve downloading and testing SH/RH apps.

Qualitative findings aligned with the *WHO Classification of Digital Health Interventions* [4]. For instance, perceptions that SRH apps may be particularly helpful for youth and persons in rural areas align with classification *1.1.2* regarding *targeted health information to clients based on demographics* [4]. Discussions regarding SRH apps for community mobilization aligns with classification *1.3.1 regarding peer groups for clients* [4]. Feedback from HCP on how digital technology can be used to link clients to health facilities and community workers is congruent with *2.4.2, remote monitoring of client health or diagnostic data by the provider*. The importance of SRH apps alongside regular reminders for health care visits aligns with *1.1.3, targeted alerts, and reminders to clients*. SRH apps that facilitate clients entering data to receive test results maps onto *1.4.2, self monitoring of diagnostic data*. Finally, HCP recommendations for online training on SRH mobile apps are relevant for *2.8.1, training content to healthcare providers*.

There are study limitations. Non-random sampling and limited survey languages limit the generalizability of findings. While we included 825 participants from 112 countries, small sample sizes per country and region preclude region-specific analyses. Due to online recruitment, the survey may disproportionately include persons with computer/internet access, persons already using digital technologies, and persons connected to SRH listservs. Despite these limitations, this is the largest to date survey on self-care SRHR interventions and generated perspectives from lay persons and HCP to inform future representative studies.

To support the increased access and use of quality digital technologies linked to self-care interventions for SRHR and healthcare systems, community mobilization and online HCP training emerged as important considerations. Digital tools can be leveraged to train HCP to support SRH mobile app usage among clients.

## Paper context

There is a dearth of knowledge regarding global perspectives from healthcare providers on how they are leveraging SRH apps in their healthcare practice. To inform the 2019 World Health Organization (WHO) consolidated guideline on self-care interventions for SRH, we conducted a global values and preferences survey. Our study revealed that nearly half of healthcare providers wanted more information and training to use SRH apps in their practice. Findings can inform SRH healthcare provider training.
